# Implementing “Online Communities” for pregnant women in times of COVID-19 for the promotion of maternal well-being and mother-to-infant bonding: a pretest–posttest study

**DOI:** 10.1186/s12884-022-04729-5

**Published:** 2022-05-18

**Authors:** Eva S. Potharst, Mirla A. Schaeffer, Cecile Gunning, Merith Cohen de Lara, Myrthe G. B. M. Boekhorst, Lianne P. Hulsbosch, Victor J. M. Pop, Sasja N. Duijff

**Affiliations:** 1 Research Institute of Child Development and Education, University of Amsterdam, Gebouw D, Roeterseilandcomplex, Nieuwe Achtergracht 127, 1018 WS Amsterdam, the Netherlands; 2 UvA Minds, Academic Outpatient (child and adolescent) Treatment Center of the University of Amsterdam, Banstraat 29, 1071 JW Amsterdam, the Netherlands; 3 Amsterdam Law and Behaviour Institute (A-LAB), Vrije Universiteit Amsterdam, De Boelelaan 1077a, 1081 HV Amsterdam, the Netherlands; 4 Netherlands Institute for the Study of Crime and Law Enforcement, De Boelelaan 1077, 1081 HV Amsterdam, the Netherlands; 5 Infant Mental Health Expertise Centre OuderKindLijn, Javastraat 155, 1095 CC Amsterdam, The Netherlands; 6 Outpatient Maternal Mental Health Practice Psyche en Zwangerschap, Cornelis Anthoniszstraat 28, 1071 VV Amsterdam, The Netherlands; 7 Department of Medical and Clinical Psychology, Center of Research On Psychological and Somatic Disorders (CoRPS), Tilburg University, Gebouw TIAS, Warandelaan 2, 5037 AB Tilburg, the Netherlands; 8 Clinical Child, Family and Education Studies, University of Utrecht, Heidelberglaan 1, Postbus 80140, 3508 TC, Utrecht, The Netherlands

**Keywords:** Pregnancy, Maternal mental health, Stress, COVID-19, Online intervention, Prenatal bonding

## Abstract

**Background:**

The Coronavirus Disease 2019 (COVID-19) pandemic elevated the risk for mental health problems in pregnant women, thereby increasing the risk for long-term negative consequences for mother and child well-being. There was an immediate need for easily accessible interventions for pregnant women experiencing elevated levels of pandemic related stress.

**Methods:**

A three-session intervention “Online Communities” (OC) was developed at the beginning of the Dutch lockdown, and implemented by a team of midwives and psychologists specialized in Infant Mental Health. Pretest (*N* = 34) and posttest (*N* = 17) measurements of depressive symptoms, worries about COVID-19 and worries in general, and mother-to-infant bonding were administered, as well as a posttest evaluation.

**Results:**

At pretest, the OC group was compared to two reference groups of pregnant women from an ongoing pregnancy cohort study: a COVID-19 (*N* = 209) and pre-COVID-19 reference group (*N* = 297). OC participants had significantly more depressive symptoms than both reference groups, and less positive feelings of bonding than the COVID-19 but not the pre-COVID-19 reference group. Compared to pretest, significant decreases in depressive symptoms (with significantly less participants scoring above cut-off) and worries about COVID-19 (large effect sizes) and worries in general (moderate to large effect size) were found at posttest for the OC participants. No significant improvement was found in bonding. Participants rated the intervention positively.

**Conclusions:**

The current study provides initial evidence supporting the idea that OC is a promising and readily accessible intervention for pregnant women experiencing stress due to the COVID-19 pandemic, and possibly also applicable to other stressors.

**Trial registration:**

This intervention was registered in the Netherlands Trial Registration (registration number Trial NL8842, registration date 18/08/2020).

## Background

The prenatal period is a very important and vulnerable period. It is a period with a huge potential for emotional growth for a mother-to-be, but also one in which she may be more vulnerable to the negative influence of environmental stressors [1]. Pregnancy increases the risk for elevated levels of stress [2], and in turn, elevated levels of stress and/or chronic stress increase the risk of maternal mental health problems during pregnancy [3] which can trigger a chain of effects. For one, prenatal stress and mental health problems in a mother can affect prenatal mother-to-infant bonding, which can be described as maternal positive feelings towards the unborn infant [4–6]. Suboptimal prenatal bonding is in turn regarded as a risk factor for suboptimal postnatal bonding [7, 8] and subsequently, suboptimal postnatal bonding is a risk factor for additional parental stress [9] and suboptimal infant development [10]. Also, maternal prenatal stress and mental health problems also increase the risk of developing maternal postpartum depression [11, 12], and child emotional problems [13]. Therefore, it is highly important that women experiencing elevated levels of stress or mental health problems during pregnancy have low threshold access to professional support [14]. The importance of low threshold, professional support for pregnant women became more critical due to more widespread distress that was related to the outbreak of the Coronavirus Disease 2019 (COVID-19) and the subsequent lockdowns which resulted in very limited access to professional support, including antenatal appointments.

COVID-19 was first reported in Wuhan, China in December 2019, quickly spreading to and within many countries, resulting in a pandemic by March 2020. Both the serious health risks of COVID-19, and the social and economic consequences were distressing. The incidence of mental health problems as a reaction to the COVID-19 crisis was high in pregnant women [15–17]. Several sources of stress for pregnant women as a result of COVID-19 have been described. One area of stress pertained to medical aspects around COVID-19, e.g. worries that COVID-19 may be a threat to their own or their baby’s life, and the fear of infection when utilizing pregnancy-related care in hospitals. Another area was centered around the available medical care: e.g. concerns about the unavailability of prenatal care, and hospital policies limiting the number of people that could accompany the women to the hospital and be present at the baby’s birth. A third area concerned more emotional areas of functioning: social isolation and problems in the relationship with their partner, increased external demands (for example pressure from a family member to take safety measures), and unavailable or contradictory information [15, 18–21].

Resilience factors have been described that seem to protect against elevated levels of stress and mental health problems in pregnant women during a pandemic. Lebel et al. [15] found that feeling socially supported was associated with lower mental health problems in pregnant women during the COVID-19 pandemic. Another study showed that pregnant women who reported a higher level of social support during the COVID-19 pandemic reported less symptoms of anxiety [22]. Jiang et al. [18] found another resilience factor, namely access to antenatal care services via hospitals’ official social media accounts. This was associated with a significantly lower risk of suffering from mental health problems, while pregnant women who received health care information from friends and family via social media had a higher risk of experiencing depression. The authors suggest that it is very important for pregnant women during a pandemic to have access to health information that is comprehensive, reliable and credible.

Several studies provided recommendations on support for pregnant women during the COVID-19 pandemic. Multiple recommendations stress the importance of developing novel methods of mental health care, such as technology-based, psychosocial interventions. Online services and/or social media platforms are discussed for fostering social support and mother-to-infant bonding [18, 19, 23, 24]. McDowell and Salvi [25] state that acceptance and mindfulness strategies of Acceptance and Commitment Therapy can help individuals cope with the challenges, uncertainties, but also opportunities that are inherent of the COVID-19 pandemic, by learning them to stay present, open up to the unpleasant feelings, and move toward valued behavior.

At the beginning of the Dutch lockdown in March 2020, a midwife and a psychologist noticed that many pregnant women had elevated levels of stress or mental health problems, and were contacting their practice with similar questions and needs, all subjects suitable for discussing in groups with other pregnant women. They initiated the development and implementation of a 3-session online intervention for pregnant women with elevated levels of stress, *Online Communities (OC).* The intervention OC was developed outside of any research project. The intervention was informed by literature on resilience factors protecting against elevated levels of stress and mental health problems in pregnant women during a pandemic, in which social support and accurate health care information from reliable sources have been found helpful for protecting against stress [15, 18, 22]. Several organizations from different regions in the Netherlands joined the project and started offering these OC to pregnant women. OC aimed to prevent further psychological dysregulation, and quickly identify and refer women that needed a more individualized and specialized intervention. The sessions existed of state-of-the-art medical updates provided by a midwife, psychoeducation on stress and motherhood by a psychologist, and group discussions and mindfulness exercises to alleviate stress.

The current pretest–posttest study evaluates the online intervention OC for pregnant women that was designed and applied in the midst of a hugely stressful period, the first months of the COVID-19 pandemic during the COVID-19 lockdown in the Netherlands. The first aim was to assess the characteristics of the pregnant women that participated in OC, as compared to other women that were pregnant during the Dutch lockdown, and also to other pregnant women before the start of the pandemic. The second aim was to assess the acceptability of OC. The third aim was to assess effectiveness of OC with regard to decreasing symptoms of depression worries about COVID-19 and worries in general, and enhancing prenatal mother-to-infant bonding.

## Methods

### Participants

In the current study, the data of three groups of women were examined. The first was a group of women that were advised by their midwife to participate in the OC intervention in different parts of the Netherlands. The participants were referred based on the midwife’s clinical impression that a mother was suffering from elevated levels of stress because of COVID-19. Examples of complaints that the participants had were rumination, insomnia, anxiety, tension, being afraid to leave the house, watching the news excessively, being afraid to give birth in the hospital, feeling isolated, worrying about the consequences of the COVID-19 crisis for the (unborn) baby and family members, and disagreement with the partner about the COVID-19 measures that needed to be taken. Of the 65 women that received an invitation, 34 women (52%) decided to participate in the study. Participants were included in the study between April 23, 2020 and June 15, 2020. Participants lived in the areas of the three participating organizations in the Netherlands, namely in the West (Amsterdam), South (Den Bosch), and East (Hengelo) of the Netherlands.

The second and third group that were used as reference groups for the first group, consisted of pregnant women already participating in a large, ongoing prospective longitudinal cohort study being carried out in the southeast of the Netherlands: the Brabant study [26]. At pretest, the OC group was compared to these reference groups: a COVID-19 reference group (*N* = 209), and a pre-COVID-19 reference group (*N* = 297). The COVID-19 reference group consisted of women who completed the third trimester measurement during the Dutch lockdown period (March 15, 2020 to August 19, 2020), and the pre-COVID-19 reference group existed of women who completed the third trimester measurement before the COVID-19 crisis (September 5, 2018 to December 30, 2019). Sociodemographic and pregnancy related characteristics of the groups are shown in Table 1.

**Table 1:** Sociodemographic and pregnancy related characteristics of the OC group and reference groups

	OC group (*N* = 34)		COVID-19 reference group (*N* = 209)		Pre-COVID-19 reference group (*N* = 297)		*F*
	Mean (SD)	Range	Mean (SD)	Range	Mean (SD)	Range	
Age	32.59 (3.66)	24–41	30.80 (3.47)	19–45	30.92 (3.73)	21–41	3.64*
Gestational age	30 (5.76)	18–40	28.06 (0.87)	26–33	28.50 (1.13)	26–38	13.35***
	*N* (%)		*N* (%)		*N* (%)		χ^2^
Living with partner	33 (97.1)		201 (98)		284 (97.3)		0.35
Level of education							21.95**
-Primary	0 (0)		0 (0)		1 (0.3)		
-Lower secondary	0 (0)		4 (2.0)		11 (3.8)		
-Higher secondary	2 (5.9)		3 (1.5)		4 (1.4)		
-Vocational	0 (0)		47 (22.9)		85 (29.1)		
-(Applied) Sciences	32 (94.1)		151 (73.7)		191 (65.4)		
Population group							7.41*
-Dutch	31 (91.2)		199 (97.1)		288 (98.6)		
-Other	3 (8.8)		6 (2.9)		4 (1.4)		
Primiparous	23 (67.6)		99 (48.3)		159 (55.2)		5.30

Differences in mean scores and proportions between the groups are analyzed using ANOVA or χ2 tests

^*^ *p* < .05, ** *p* < .01, *** *p* < .001

### Procedures

**Fig. 1:**
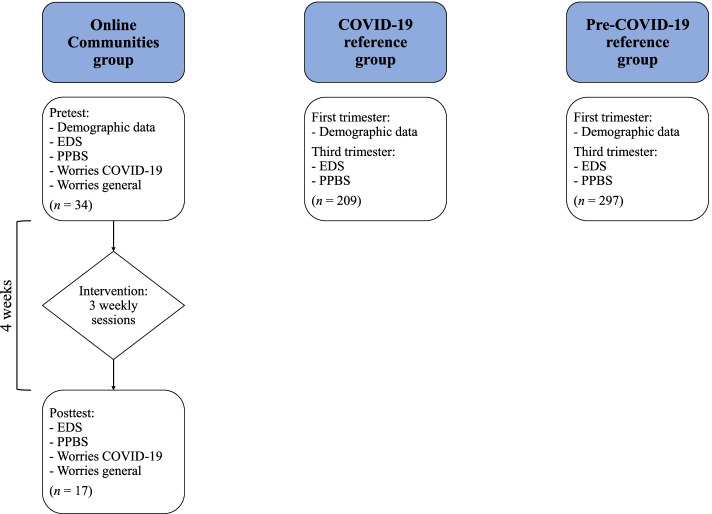
Schematic overview of the current study

A schematic overview of the study design can be found in Fig. 1. The current study used a pretest–posttest design. This intervention was registered in the Netherlands Trial Registration (registration number Trial NL8842, registration date 18/08/2020). The ethical committee of the Faculty of Social and Behavioral Sciences of the University of Amsterdam approved the study (2020-CDE-12134). The OC participants gave informed consent via an online form, before completing the first set of online questionnaires. The ethical committee gave permission for online instead of written informed consent, because this seemed more suitable given the situation at the time (lockdown). The Medical Ethics Committee at the Máxima Medical Centre in Veldhoven approved the Brabant Study (L64091.015.17). The Brabant Study participants provided written informed consent.

All mothers who were referred to an OC group were sent an email with practical information by one of the three participating organizations. This email also included an invitation to participate in the OC research project, and an information letter about the project. Participants could click on a link in the email that first led to an informed consent form, and then to the pretest questionnaires. Participants were asked to fill out their email address, so that they could be contacted again for posttest measurement. The intervention lasted three weeks. Four weeks after participants had completed the pretest questionnaires, they were invited via email to complete the posttest measurement. When participants did not respond to this posttest invitation, they were invited twice more via email.

The data of the reference groups was drawn from an ongoing large prospective longitudinal study investigation the psychological and physiological wellbeing among women who are followed from pregnancy until the early postpartum; the Brabant Study [26]. Community midwives and hospitals in the southeast of the Netherlands recruited participants. Recruitment started in 2019. Women who were older than 18 and who had a sufficient understanding of the Dutch language could be included in the Brabant Study. Exclusion criteria were multiple pregnancy, endocrine disorder prior to pregnancy (other than thyroid function problems), diabetes type I, rheumatoid arthritis, severe psychiatric disorder (schizophrenia, borderline personality disorder or bipolar disorder), HIV, drug or alcohol addiction problems or any other disease resulting in treatment with drugs that can be potentially harmful to the fetus. During pregnancy, participants complete online questionnaires in all three trimesters (12, 20, 28 weeks) of their pregnancy and at 8–10 weeks postpartum. For the current study, data was used from the first measurement (sociodemographic data) and the third measurement, when mothers were in their third trimester of pregnancy (data on prenatal mother-to-infant bonding and depressive symptoms).

### Intervention

The intervention consisted of three weekly 90-min online sessions, hosted and moderated by a psychologist specialized in Infant Mental Health (IMH) (among others SD and CG) and/ or specialized in mental health during pregnancy (among others MCL), and co-hosted by a midwife. The content of the sessions is listed in Table 2. A maximum of 14 pregnant women participated in each group. The content of the sessions was eclectic, but primarily based on the IMH framework, a framework that recognizes the importance of the developing relationship between a mother and her unborn child, the role that stress plays in the developing parent–child relationship, how not only parental and child factors, but also environmental factors shape the parent–child relationship, and focuses on what is needed to optimize the (developing) parent–child relationship [27]. Interventions based on the IMH framework that were used during the sessions were: an information sheet with an explanation about stress, massage exercises and references to YouTube video’s with examples of how to massage the pregnant belly, tips for the development of a bond with the (unborn) baby, explanation on ‘It takes a village to raise a child’, and psychoeducation on the fantasy baby, phantom baby and actual baby. Furthermore, techniques from Cognitive Behavioral Therapy were used, such as structuring your day, weekly agenda, structuring and limiting the time spent on reading and watching news (news diet and news time), and alternatives for activities that you used to undertake. Also techniques from Acceptance and Commitment Therapy were used, namely psychoeducation about ‘undesirable emotions’ and rumination, the acceptance of sadness and fear, explanations about how to talk about feelings to others, and a video on empathy versus sympathy by Brené Brown. Also conversation techniques from Centuring Pregnancy were used (for example the question ‘What tip from last time did you benefit from, and would you like to share?). Every session started with a state-of-the-art medical update provided by the midwife and an inventory of all the pertinent questions that the women had for the midwife and/ or psychologist. Medical questions that could not be answered at that time were forwarded to a doctor in between sessions and were answered in the next session. After answering the questions, psychoeducation was given by the psychologist, and there was time for group discussion and sharing of experiences by the participants. Each session ended with a mindfulness exercise, namely a customized 3-min breathing space [28]. In this meditation, participants were not only invited to become aware of their inner experience, but also to verbalize this by talking to their unborn baby, e.g. ‘mommy is a bit anxious right now, but this has nothing to do with you.’ The clinical impression of the psychologists was that mothers-to-be were often touched by this exercise, and relieved to be offered tools in how to deal with stress without it affecting their baby. The psychologist offered psychoeducation on people’s tendencies to ignore difficult feelings, and the negative effect of stress on an unborn baby, and how mothers-to-be could help both themselves and their children (both now as well as in the future) by learning to recognize, deal with and give words to their feelings. Other themes that were discussed included the Circle of Security [29], the Window of Tolerance [30], the importance of a daily structure and physical movement, and social contact (and the building of a bond between the expected baby and the extended family) in times of lockdown.

**Table 2:** Outline of the three Online Communities sessions

**Session 1. Dealing with anxiety and stress in times of corona** Welcome by host • Introduction: - Host & co-host (occupation & background). Explain Online Communities & zoom - Participants: name, due date and invite to ask something, what is on their mind now (Write this question in the chat function) Midwife: • Summarize and answer questions Psychologist: • Explanation of stress and how you can influence stress • Group discussion Closing round • How did you experience it? What else would you like to know? Will you be there next time? Mindfulness exercise • The three minute breathing space
**Session 2. Stress and the Window of Tolerance** Welcome by host • How did you experience it last time? How did it go this week? Do you have any questions? Midwife: • Communicate last week's changes in protocols • Answer the questions left unanswered last week • Answer the new questions • Provides an overview of the latest Covid developments in birth care Psychologist: • Window of tolerance psycho education • Explanation of accepting emotions and how they can co-exist (eg you can be afraid of Covid and at the same time be happy your partner is working at home) - Group discussion on: What bothers you most right now? What do you miss the most right now? What is positive about this time? Did anyone use tips from last week and how was that? - Facilitate a group discussion on sadness, loneliness and - Invite them to concrete exercises to make room for your child, make an alternative birth plan. Many women avoid thinking out of fear. Explain what that does. Let them exchange concrete tips Closing round: • Round with questions Mindfulness exercise • The three minute breathing space with your baby
**Session 3. Dealing with situations that you cannot change** Welcome by host • How did you experience it last time? How did it go this week? Do you have any questions? Midwife: • Communicate last week's changes in protocols • Answer the questions left unanswered last week • Answer the new questions • Provides an overview of the latest Covid developments in birth care Psychologist: • Fantasizing about being pregnant and then having a baby during Corona • The importance of structure & news diet • Making time for your baby (prenatal) • Self-care and caring for a partner Closing round: • Evaluation. How do you proceed? What tips do you take with you Mindfulness exercise: •The three minute breathing space with your baby

### Measures

Symptoms of depression were measured using the Edinburgh Depression Scale (EDS) [31]. The EDS consists of 10 items that are scored on a scale from 0 to 3. Total scores range from 0 to 30, with higher scores indicating more symptoms of depression. A cut-off point specific for the second and third trimester was used (≥ 10) and for the postpartum period (≥ 13) if parturition occurred between pretest and posttest in the OC group. This instrument and cut-off point are validated in pregnant women from a Dutch sample [32]. Cronbach’s alpha in the current study were 0.89 at pretest for the OC group, and 0.86 and 0.87 for the COVID-19 and pre-COVID-19 reference groups, respectively.

Prenatal mother-to-infant bonding was measured using the prenatal version of the Pre- and Postnatal Bonding Scale (PPBS) [33]. Five positively formulated items were scored on a 4-point Likert scale. Total scores range between 0 and 15 with higher scores meaning more positive feelings of bonding. The scale has shown to be reliable and valid in a Dutch sample of pregnant women [33]. Cronbach’s alpha in the current study were 0.84 at pretest for the OC group, and 0.86 and 0.91 for the COVID-19 and pre-COVID-19 reference groups, respectively.

Worries about COVID-19 and worries in general (about things unrelated to COVID-19) were measured using two single items about the extent to which the participant had worries, that were answered on a scale from 0 to 10, with higher scores indicating more worries. The first item was: ‘how severe are your worries concerning COVID-19 at the moment?’, and the second item: ‘how severe are your worries related to other things at the moment?’.

Questions about sociodemographic and pregnancy related variables were answered online at pretest by the OC group, and at the first trimester measurement by the reference groups. The following variables were included in the study: maternal age (in years), level of education (primary, lower secondary, higher secondary, vocational, or (applied) sciences), population group (Dutch or different), marital status (married or living together, divorced, living apart together or single), gestational age (in weeks), and parity (para 0, 1, 2, or ≥ 3).

Questions evaluating the training were completed at posttest for the OC groups. Questions either were close-ended or answered with a 5-point Likert scale. The specific questions that were asked are listed in Table 3.

**Table 3:** Evaluation of OC at posttest (*n* = 17)

Questions/statements			Yes	No	Not applicable
Did OC meet your expectations?			16 (94.1%)	1 (5.9%)	0 (0%)
I was able to ask all my questions and express my worries during the sessions			14 (100%)	0 (0%)	0 (0%)
I am planning to stay in touch with the other participants			4 (23.5%)	13 (76.5%)	0 (0%)
	Completely disagree	Disagree	Agree	Completely agree	Not applicable
I felt supported during the sessions	0 (0%)	0 (0%)	6 (35.3%)	10 (58.8%)	1 (5.9%)
The following elements were useful for me:					
Evaluation of OC at posttest - Information given by the midwife	0 (0%)	0(0%)	9 (52.9%)	8 (47.1%)	0 (0%)
- Information given by the psychologist	0 (0%)	0 (0%)	7 (41.2%)	10 (58.8%)	0 (0%)
- Group discussions	0 (0%)	2 (11.8%)	6 (35.3%)	6 (35.3%)	3 (17.6%)
-Sharing experiences	0 (0%)	2 (11.8%)	7 (41.2%)	8 (47.1%)	0 (0%)

Data are presented as *n* (%)

### Data analysis

Differences in categorical sociodemographic characteristics between the OC group and the reference groups were assessed using contingency table analyses test, with pairwise post hoc analyses if significant. For continuous characteristics an ANOVA was used with Tukey’s post hoc tests if significant, or Welch’s ANOVA with Games-Howell post hoc tests if Levene’s test indicated unequal variances, which was the case for gestational age (*F* = 225.49, *p* < 0.001).

Differences between the OC group and the reference groups in depressive symptoms and bonding were analyzed using ANOVA with planned contrast, or Welch’s ANOVA with planned contrast if Levene’s test indicated unequal variances, which was the case for bonding (*F* = 3.15, *p* = 0.044). A contingency table analyses test, with post hoc pairwise comparisons if significant, was used to assess differences between the three groups on the proportions of participants scoring above the cut-off on the EDS.

Pretest versus posttest differences were analyzed using paired sample t-tests, or with Wilcoxon-signed rank test if Shapiro–Wilk’s test the pretest–posttest difference scores were non-normally distributed, which was the case for bonding (*D*(17) = 0.81, *p* = 0.002). A McNemar test was used to assess a possible difference in the percentage of participants experiencing depressive symptoms above the before mentioned cut-off scores. For all main effects a Pearson’s correlation coefficient, *r*, was calculated. Effect sizes were interpreted as small (*r* ≥ 0.1), moderate (*r* ≥ 0.3), and large (*r* ≥ 0.5) [34]. Effects were regarded significant when *p* < 0.05.

A post hoc power analysis was conducted using the software G*Power [35]. For the calculation, a matched pairs t-test and an alpha error probability of 0.05 was selected. Furthermore, we used the effect sizes that were found. Power ranged between 0.66 and 0.82.

## Results

### Response rate

Of the 34 OC participants that completed a pretest assessment, 17 (50%) completed the posttest assessment. In the posttest assessment, 4 of the 17 participants (24%) reported to have given birth. Data from all 34 participants were used when comparing the OC group with the reference groups, while only data from the 17 participants that completed the posttest assessment were used to assess the acceptability and effectiveness of the OC intervention. There were no differences in pretest characteristics between the participants that did and did not complete the posttest measurement.

### Characteristics of OC participants

**Fig. 2:**
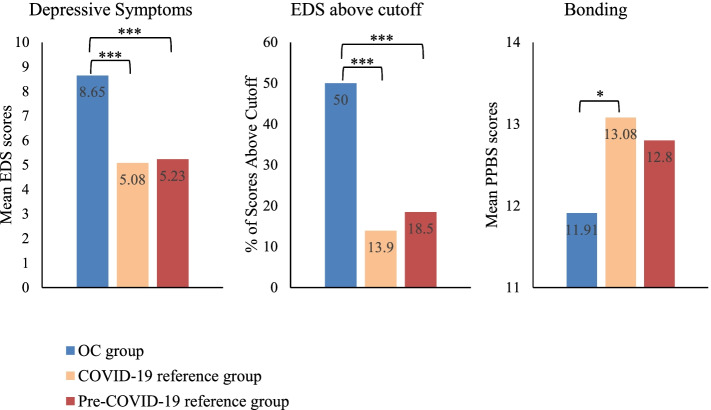
Characteristics of OC participants and reference groups. *p ≤ .050. ***p ≤ .001

Differences between the groups were found for age, gestational age, level of education and population group (see Table 1). Furthermore, ANOVA analyses showed a significant difference between groups both on depressive symptoms, *F*(2, 529) = 9.25, *p* < 0.001, and on prenatal mother-to-infant bonding, *F*(2,89.87) = 3.18, *p* = 0.046. Planned contrast revealed no significant difference between the pre-COVID-19 and COVID-19 reference groups on either depressive symptoms (*M* = 5.23, *SD* = 4.56 and *M* = 5.08, *SD* = 4.53) or bonding (*M* = 12.80, *SD* = 2.51 and *M* = 13.08, *SD* = 2.20). However, the OC groups scored significantly higher on depressive symptoms (*M* = 8.65, *SD* = 5.07) than the pre-COVID-19 reference group, *t*(529) = 4.12, *p* < 0.001, *r* = 0.18, as well as the COVID-19 reference group, *t*(529) = 4.21, *p* < 0.001, *r* = 0.18 (small effect sizes). Concerning bonding, the OC group scored significantly lower (*M* = 11.91, *SD* = 2.69) than the COVID-19 reference group, *t*(40.61) =  − 2.40, *p* = 0.021, *r* = 0.35, (moderate effect size), but not the pre-COVID-19 reference group, *t*(39.96) =  − 1.83, *p* = 0.075, *r* = 0.28, (small to moderate effect size). Furthermore, there was a significant difference between groups in the proportion of participants scoring above the EDS cutoff, χ^2^(2, *N* = 532) = 24.19, *p* < 0.001. Post hoc pairwise comparisons indicated that while there was no significant difference between the reference groups, the proportion of participants above the cut-off was larger in the OC group than in both the pre-COVID-19 and the COVID-19 reference group, χ^2^(1, *N* = 327) = 16.76, *p* < 0.001 and χ^2^(1, *N* = 239) = 24.12, *p* < 0.001 respectively. Differences between the groups on the outcome measures are shown in Fig. 2.

### Acceptability of OC

The results of the posttest evaluation questionnaire are shown in Table 3. Of the 17 participants that completed the posttest measurement, 16 participants (93%) answered ‘yes’ to the question whether OC had met their expectations. The only participant that answered ‘no’ to this question, specified that she stopped following OC after the first session, because she already received a lot of support from her midwife and psychologist. Participants were asked to rate how helpful OC was for them on a scale from 0 to 10, and scored an 8.1 on average (SD = 0.7).

### Effectiveness of OC

**Fig. 3:**
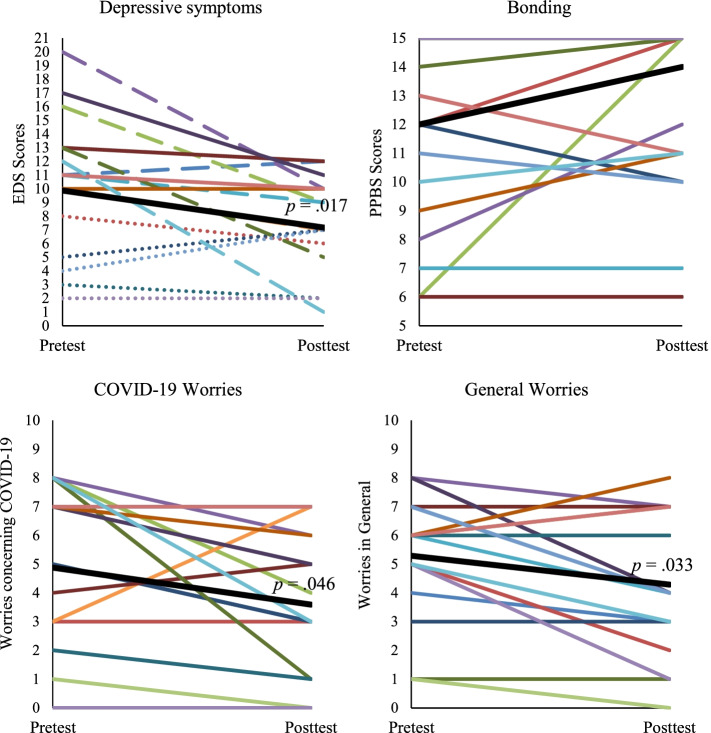
Effectiveness of Online Communities per participant. *Note.* Thick black lines indicate the group mean. For Depressive Symptoms: dotted lines indicate the participant never scored above clinical cutoff, striped lines indicate the participant scored above clinical cutoff during pretest but not posttest, and solid lines indicate the participant scored above clinical cutoff during both pre- and posttest

A paired samples T-test indicated a significant decrease in depressive symptoms from pre- (*M* = 9.88, *SD* = 5.31) to posttest (*M* = 7.18, *SD* = 3.68), *t*(16) = 2.67, *p* = 0.017, *d* = 0.65, (moderate effect size). A McNemar test showed that the proportions of participants scoring above the cutoff for depressive symptoms were different, with less participants scoring above cutoff at posttest (*N* = 4, 23.5%) compared to pretest (*N* = 11, 64.7%), *p* = 0.016 (2 sided). Although levels of bonding increased between pre- (*Mdn* = 12) and posttest (*Mdn* = 14), a Wilcoxon-signed rank test indicated this difference was not significant, *z* =  − 1.49, *p* = 0.136, *r* =  − 0.26 (small to moderate effect size). Paired samples T-tests indicated a significant decrease in worries concerning COVID-19 from pre- (*M* = 4.88, *SD* = 2.91) to posttest (*M* = 3.59, *SD* = 2.55), *t*(16) = 2.16, *p* = 0.046, *d* = 0.52 (moderate effect size), and in worries in general from pre- (*M* = 5.29, *SD* = 2.05) to posttest (*M* = 4.29, *SD* = 2.50), *t*(16) = 2.33, *p* = 0.033, *d* = 0.57 (moderate effect size). Figure 3 depicts pretest and posttest scores of the individual participants on all outcomes, and mean pretest and posttest scores of the OC group on all outcomes.

## Discussion

The current study evaluated OC, an online intervention developed for Dutch pregnant women experiencing stress related to the COVID-19 pandemic. The main findings of the current study were that the women that participated in OC during the Dutch lockdown had higher levels of depressive symptoms and lower mother-to-infant bonding scores than women from a general population that were in the third trimester of their pregnancy during the COVID-19 pandemic. They also had higher levels of depressive symptoms (but not lower mother-to-infant bonding scores) than women from a general population that were in the third trimester of their pregnancy before the breakout of the COVID-19 pandemic. The participants that completed posttest after following the intervention (50%), reported a decrease in depressive symptoms and worries about COVID-19 and worries in general (moderate effect sizes), and rated the intervention positively. The improvement in mother-to-infant bonding from pretest to posttest as reported by these participants was non-significant.

Inclusion of participants in the OC intervention was based on midwives’ clinical impressions. The results of this study confirmed that the participants that were selected for the intervention indeed showed more depressive symptoms than the general population of women that were pregnant during the COVID-19 pandemic. Half of the participants scored above the cut-off for elevated depressive symptoms during the pretest assessment, while this was less than 20% for the reference groups. We also found a difference in mother-to-infant bonding between the OC group and the COVID-19 reference group, with OC participants reporting less positive feelings of bonding. This showed that the women in the current study were in need of support, focused on both their personal mental health, but also Infant Mental Health support, focused on improving the developing relationship between a pregnant mother and her (unborn) baby. The need to focus on both the mother’s mental health and on the developing relationship between the mother and baby has especially been demonstrated by research on depression and difficulties in parenting and the parent–child relationship in the postnatal period. These studies showed that therapy that only focuses on mother’s mental health problems, does not improve parenting difficulties and parent–child relationship [36, 37]. The importance of taking mother-to-infant bonding into account in psychological interventions for pregnant women was further emphasized in research showing that prenatal bonding is predictive of postnatal bonding [7, 9].

We found no differences in symptoms of depression between the COVID-19 reference group and the pre-COVID-19 reference group. This is not in line with previous research on symptoms of depression in pregnant women during the COVID-19 pandemic [15–18, 37]. Most previous studies on mental health problems in pregnant women during the COVID-19 pandemic, compared the study group to standardized norm data or data from a meta-analysis. In the current study, one group completed the questionnaires before the COVID-19 pandemic and one group during the COVID-19 pandemic. A possible explanation of the fact that no difference was found on symptoms of depression between the COVID-19 and pre-COVID-19 reference group is that the measures and restrictions that were enforced by the Dutch government were different from the restrictions in most of the countries that the other studies were conducted in. The Dutch government did not decide on a full lockdown, but instead on a so-called ‘intelligent lockdown’, which allowed for more activities for the residents [38]. Possibly, the fact that people were allowed to move around freely alone or as a family (e.g. for walks and work-out), or meet a maximum of three people outside of one’s direct household while keeping a distance of 1.50 m, may have had a positive effect on symptoms of depression in pregnant women. Lebel et al. [15] indeed found that both physical activity and social support made pregnant women more resilient against mental health problems.

Results on the acceptability of the intervention showed that the participants who completed the posttest rated the intervention positively. Most of these participants experienced the group discussions and sharing of experiences between the participants as helpful. In addition, participants who completed the evaluation form experienced the information given by the midwife and the psychologist as helpful. This in line with research of Jiang et al. [18] that showed that pregnant women were in need of validated information from professionals during the COVID-19 pandemic.

With regard to the effectiveness of OC, the results of this study showed that for the participants who completed posttest (50%), it was effective in decreasing symptoms of depression and worries about COVID-19 and worries in general. Although OC consists of fewer sessions than most other online interventions, the results are in line with a systematic review on internet-based mental health interventions, showing that online interventions can indeed alleviate of symptoms of depression and anxiety [39]. However, no significant improvement in prenatal mother-to-infant bonding was found. Possibly, a short, online intervention is not sufficient to improve bonding. An alternative explanation is that power was too limited to show a significant difference between pretest and posttest. Previous research on other interventions and strategies aimed at improving parental bonding to the unborn baby also failed to show positive effects [40]. A narrative review on parental bonding to the unborn baby, including 27 papers, found that results were inconsistent due to the large diversity of interventions and variation in methodological quality [40]. Therefore, it was concluded that there is insufficient evidence regarding the effectivity regarding any included intervention.

Strengths of the current study were the inclusion of reference groups, and the fact that we were able to develop an intervention and start a study to evaluate it within a month after the start of the Dutch lockdown. There are some limitations to take into consideration. First, the reference groups that were used for this study were not completely comparable to the OC group with regard to the level of education and other sociodemographic characteristics, and the moment in pregnancy that the questionnaires were completed. It may be that these differences accounted for (part of the) baseline difference in depressive symptoms and bonding between the OC group and the reference groups. Also, the reference group did not complete both measurements. It is therefore not possible to compare improvement over time with a control group, which limits the conclusions that can be drawn on the beneficial effects of the intervention. Furthermore, the statistical analysis did not take into account possible confounding variables, such as changes in medication or social support outside the intervention. Therefore, it is not clear whether such variables may have played a role in the improvements that were shown. Another limitation is associated with the limited percentage of women who participated in the intervention who decided to also partake in the study (52%), and the low response rate at posttest of the women who completed pretest (50%). This means that effects of the intervention could be studied in only a quarter of the participants. Because the characteristics of a large part of the intervention-participants are unknown, it is also unknown whether the results of the current study are generalizable. Lastly, there may be hindrances in the implementation of this intervention in other countries than the Netherlands, because of the differences in the organization of the health care system for pregnant women, with generally a smaller role for midwifes.

Future studies could include longer-term follow-up, especially in the postnatal period, could shed light on the question whether OC might play a role in preventing postnatal maternal mental health problems and suboptimal mother-to-infant bonding. Furthermore, it could be studied whether selection of possible participants for OC could be improved by using screening with use of questionnaires. Future studies could also examine whether an (adjusted version of) OC may also be feasible, acceptable and effective for groups of pregnant women that experience different sources of stress than a pandemic, such as obstetric problems.

## Conclusions

In conclusion, the current study provides initial evidence that the online intervention ‘Online Communities’ is a promising online intervention for pregnant women experiencing stress as a result of the COVID-19 pandemic. Offering OC to this specific group promotes maternal mental health by decreasing depressive symptoms and worries about COVID-19 and worries in general in mothers-to-be. OC participants who completed the evaluation (50%) rated the intervention positively. The online character of the intervention makes it readily accessible for large groups of women with no travel required, thereby making it safe in terms of physical distancing, and it is cost-effective. This is an important outcome, as it is still unsure when the pandemic will end. Possibly, adjusted versions of OC may also be suitable for pregnant women experiencing stress related to other circumstances.

## Data Availability

The datasets used and/or analysed during the current study are available from the corresponding author on reasonable request.

## Abbreviations

OC: Online Communities
EDS: Edinburgh Depression Scale
PPBS: Pre-and Postnatal Bonding Scale
COVID-19: Coronavirus Disease 2019

## References

[1] Bales M, Pambrun E, Melchior M, Glangeaud-Freudenthal NC, Charles MA, Verdoux H (2015). Prenatal psychological distress and access to mental health care in the ELFE cohort. Eur Psychiatry.

[2] Woods SM, Melville JL, Guo Y, Fan MY, Gavin A (2010). Psychosocial stress during pregnancy. Am J Obstet Gynecol.

[3] Dayan J, Creveuil C, Dreyfus M, Herlicoviez M, Baleyte JM, O'Keane V (2010). Developmental model of depression applied to prenatal depression: role of present and past life events, past emotional disorders and pregnancy stress. PLoS One.

[4] Figueiredo B, Costa R (2009). Mother’s stress, mood and emotional involvement with the infant: 3 months before and 3 months after childbirth. Arch Women Ment Health.

[5] Maas AJB, Vreeswijk CM, Braeken J, Vingerhoets AJ, van Bakel HJ (2014). Determinants of maternal fetal attachment in women from a community-based sample. J Reprod Infant Psychol.

[6] Dubber S, Reck C, Müller M, Gawlik S (2015). Postpartum bonding: the role of perinatal depression, anxiety and maternal–fetal bonding during pregnancy. Arch Womens Ment Health.

[7] Cuijlits I, van de Wetering AP, Endendijk JJ, van Baar AL, Potharst ES, Pop VJM (2019). Risk and protective factors for pre-and postnatal bonding. Infant Ment Health J.

[8] de Cock ES, Henrichs J, Vreeswijk CM, Maas AJ, Rijk CH, van Bakel HJ (2016). Continuous feelings of love? The parental bond from pregnancy to toddlerhood. J Fam Psychol.

[9] de Cock ES, Henrichs J, Klimstra TA, Maas AJB, Vreeswijk CM, Meeus WH, van Bakel HJ (2017). Longitudinal associations between parental bonding, parenting stress, and executive functioning in toddlerhood. J Child Fam Stud.

[10] le Bas GA, Youssef GJ, Macdonald JA, Rossen L, Teague SJ, Kothe EJ (2020). The role of antenatal and postnatal maternal bonding in infant development: a systematic review and meta-analysis. Soc Dev.

[11] Bunevicius R, Kusminskas L, Bunevicius A, Nadisauskiene RJ, Jureniene K, Pop VJ (2009). Psychosocial risk factors for depression during pregnancy. Acta Obstet Gyn Scan.

[12] Yim IS, Stapleton LRT, Guardino CM, Hahn-Holbrook J, Schetter CD (2015). Biological and psychosocial predictors of postpartum depression: systematic review and call for integration. Ann R Clin Psychol.

[13] Hentges RF, Graham SA, Plamondon A, Tough S, Madigan S (2019). A developmental cascade from prenatal stress to child internalizing and externalizing problems. J Pediatr Psychol.

[14] Glover V (2014). Maternal depression, anxiety and stress during pregnancy and child outcome; what needs to be done. Best Pract Res Clin Obstet Gynaecol.

[15] Lebel C, MacKinnon A, Bagshawe M, Tomfohr-Madsen L, Giesbrecht G (2020). Elevated depression and anxiety symptoms among pregnant individuals during the COVID-19 pandemic. J Affect Disord.

[16] Ceulemans M, Hompes T, Foulon V (2020). Mental health status of pregnant and breastfeeding women during the COVID-19 pandemic: A call for action. Intl J Gynecol Obstet.

[17] Wu Y, Zhang C, Liu H, Duan C, Li C, Fan J (2020). Perinatal depressive and anxiety symptoms of pregnant women along with COVID-19 outbreak in China. Am J Obstet Gynecol.

[18] Jiang H, Jin L, Qian X, Xiong X, La X, Chen W (2020). Evidence of accessing antenatal care information via social media platforms supports mental wellbeing in COVID-19 epidemic. Bull World Health Organ.

[19] Shorey S, Chan V (2020). Lessons from past epidemics and pandemics and a way forward for pregnant women, midwives and nurses during COVID-19 and beyond: a meta-synthesis. Midwifery.

[20] Groulx T, Bagshawe M, Giesbrecht G, Tomfohr-Madsen L, Hetherington E, Lebel CA (2021). Prenatal care disruptions and associations with maternal mental health during the COVID-19 pandemic. Front Glob Womens Health.

[21] Sherin M, Gildner TE, Thayer ZM (2021). COVID-19-related changes to pregnant people's work-plans increase prenatal depression. Front Glob Womens Health.

[22] Yue C, Liu C, Wang J, Zhang M, Wu H, Li C (2020). Association between social support and anxiety among pregnant women in the third trimester during the coronavirus disease 2019 (COVID-19) epidemic in Qingdao, China: the mediating effect of risk perception. Int J Soc Psychiatry.

[23] Rajkumar RP (2020). COVID-19 and mental health: a review of the existing literature. Asian J Psychiatr.

[24] Choi KR, Records K, Low LK, Alhusen JL, Kenner C, Bloch JR (2020). Promotion of maternal-infant mental health and trauma-informed care during the COVID-19 pandemic. J Ob Gyn Neonatal Nursing.

[25] McDowell MJ, Salvi JD (2020). Casting light from the shadows: coping and defenses amidst a pandemic. J Clin Psychiarty.

[26] Meems M, Hulsbosch L, Riem M, Meyers C, Pronk T, Broeren M (2020). The Brabant study: design of a large prospective perinatal cohort study among pregnant women investigating obstetric outcome from a biopsychosocial perspective. BMJ Open.

[27] Rexwinkel M, Schmeets M, Pannevis C, Derkx B (2011). Handboek infant mental health.

[28] Segal ZV, Williams JMG, Teasdale JD (2002). Mindfulness-based cognitive therapy for depression: a new approach to relapse prevention.

[29] Powell B, Cooper G, Hoffmann K, Marvin B (2013). The circle of security intervention.

[30] Siegel D (1999). The developing mind: toward a neurobiology of interpersonal experience.

[31] Cox JL, Holden JM, Sagovsky R (1987). Detection of postnatal depression: development of the 10-item Edinburgh postnatal depression scale. Brit J Psychiatry.

[32] Bergink V, Kooistra L, van den LambregtseBerg MP, Wijnen H, Bunevicius R, Van Baar A (2011). Validation of the Edinburgh depression scale during pregnancy. J Psychosomatic Res.

[33] Cuijlits I, Wetering van de AP, Potharst ES, Truijens SEM, van Baar AL, Pop VJM. Development of a Pre-and Postnatal Bonding Scale (PPBS). J Psychol Psychother.Res. 2016;6(5):1000282. 10.4172/2161-0487.1000282.

[34] Cohen J (1992). A power primer. Psychol Bull.

[35] Faul F, Erdfelder E, Lang AG, Buchner A (2007). G*Power 3: a flexible statistical power analysis program for the social, behavioral, and biomedical sciences. Behav Res Meth.

[36] Kersten-Alvarez LE, Hosman CM, Riksen-Walraven JM, van Doesum K, Hoefnagels C (2011). Which preventive interventions effectively enhance depressed mothers' sensitivity? A meta-analysis. Infant Ment Health J.

[37] Murray L, Cooper P, Fearon P (2014). Parenting difficulties and postnatal depression: implications for primary healthcare assessment and intervention. Community Pract.

[38] Kuiper ME, de Bruijn AL, Reinders Folmer C, Olthuis E, Brownlee M, Kooistra EB, et al. The intelligent lockdown: compliance with COVID-19 mitigation measures in the Netherlands. Amsterdam Law School Research Paper. 2020. 10.2139/ssrn.3598215.

[39] Saddichha S, Al-Desouki M, Lamia A, Linden IA, Krausz M (2014). Online interventions for depression and anxiety–a systematic review. Health Psychol Behav Med.

[40] Cunen NB, Jomeen J, Xuereb RB, Poat A (2017). A narrative review of interventions addressing the parental–fetal relationship. Women Birth.

